# Dynamic Change of Quality During the Stuffed Bun Steamed Process

**DOI:** 10.3390/foods15040615

**Published:** 2026-02-08

**Authors:** Miao Bai, Haiyong Pan, Shujun Li, Wenting Zheng, Dingnan Zhang, He Liu, Lina Yang, Danshi Zhu

**Affiliations:** College of Food Science and Technology, Bohai University, Jinzhou 121013, China; baimiao@qymail.bhu.edu.cn (M.B.); 2022015126@qymail.bhu.edu.cn (H.P.); 2025015119@bhu.edu.cn (S.L.); zwt2023015199@bhu.edu.cn (W.Z.); 2022015051@qymail.bhu.edu.cn (D.Z.); liuhe@bhu.edu.cn (H.L.); yanglina@qymail.bhu.edu.cn (L.Y.)

**Keywords:** temperature, simulation, stuffed bun, quality, characteristic index, monitoring

## Abstract

The steaming process is crucial for the production of stuffed buns. This study aimed to monitor quality changes in stuffed buns during steaming and to simulate internal temperature evolution using numerical modeling, to support intelligent process control. Multiple quality attributes were evaluated during steaming, and internal temperature distributions were monitored at the bun center and at radial distances of 1, 2, and 3 cm from the center. A numerical temperature model was established and validated by comparison with experimental measurements. The results showed that most quality indicators exhibited the most pronounced changes during the initial 0–9 min of the steaming process. Among the evaluated parameters, internal temperature was identified as the most suitable indicator for monitoring the steaming state of stuffed buns. The consistency between simulated and experimental temperature profiles further confirmed the feasibility of the proposed temperature-based monitoring approach. This study provides a theoretical and technical basis for the intelligent monitoring and control of stuffed bun steaming.

## 1. Introduction

Cooked stuffed buns (baozi) are one of the staple foods in China and occupy an important place in Chinese food culture. Baozi are widely appreciated for their nutritional value, convenience, and palatability. With the globalization of food culture, baozi have gradually gained popularity in Southeast Asian countries such as Indonesia, Thailand, and Japan, and have also been increasingly accepted in Western countries [1]. At present, baozi production has been largely industrialized. The manufacturing process of stuffed buns is similar to that of steamed bread, except that fermentation is performed after filling [2]. Well-cooked baozi are characterized by a rich and flavorful filling, a glossy yet not overly thick crust, and a light, fluffy texture, which differs markedly from baked products such as Western-style toast. Moreover, steaming avoids the Maillard reaction that occurs at high baking temperatures, thereby reducing the formation of potentially harmful compounds such as acrylamide and furans [3]. In addition, the use of diverse fillings further enhances the nutritional value and sensory quality of baozi [4].

The quality of baozi is influenced by multiple factors, including ingredient quality, formulation, and processing conditions [5,6]. In current industrial practice, the determination of doneness largely relies on operators’ experience, typically by controlling steaming time and ensuring that the center temperature reaches approximately 90 °C. However, for stuffed buns with different fillings, the required steaming time may vary even when the same center temperature is achieved [7,8]. These uncertainties can reduce product consistency during industrial production, leading to increased costs and resource waste. Existing studies mainly focus on quality changes in frozen dough or re-steamed buns during storage [3,9,10], whereas systematic investigations into the dynamic quality changes of fresh baozi during steaming remain limited.

Similar to the evolution of bread baking in Western countries, the industrialization of steamed bun production is expected to become an important development trend. Meanwhile, rapid advances in artificial intelligence (AI) and intelligent manufacturing technologies have created new opportunities for process control and quality monitoring in food production.

In recent years, the food processing industry has increasingly adopted process monitoring technologies that integrate smart sensing techniques with chemometrics, enabling real-time and non-destructive quality evaluation. These approaches include machine learning, spectroscopy, numerical simulation, and electronic nose technologies, and have been successfully applied to the monitoring of fruit ripening [11], food packaging [12], steamed bread dough fermentation [13], and alcoholic beverages [14]. Effective monitoring models generally require the integration of sensor-derived signals with physical, chemical, or sensory quality indicators.

Therefore, the objective of this study was to investigate the dynamic changes of multiple quality parameters—including water distribution and mobility, starch gelatinization characteristics, specific volume, and internal temperature—during the steaming of baozi. Based on these results, suitable characteristic indicators were identified and used to establish a monitoring model for assessing the maturity state of stuffed buns during steaming. The findings of this study provide a theoretical basis for intelligent process control and contribute to the industrialization and future development of steamed bun production.

## 2. Materials and Methods

### 2.1. Materials

Highly active dry yeast (Angel^®^, Angel Yeast Co., Ltd., Yichang, China), medium gluten wheat flour (Wu deli^®^, Wu deli Co., Ltd., Baoding, China), Glycolytic enzyme (microbial α-amylase, 100 U/mL, Wanbang Chemical Technology Co., Ltd., Zhengzhou, China), Sodium hydroxide standard solution, sodium thiosulfate standard solution (aladdin^®^, aladdin Co., Ltd., Shanghai, China), Sulfuric acid, hydrochloric acid (Jinzhou Gu Cheng Chemical Reagent Co., Ltd., Jinzhou, China), and Fine sugar, chicken essence, monosodium glutamate, salt, sesame oil, ginger, and garlic juice, baking soda, light soy sauce, dark soy sauce, oyster sauce, five-spice powder, pork were purchased from a local supermarket.

### 2.2. Sample Preparation

To ensure consistency across all experiments, the bun formulation and preparation were standardized. The dough was prepared using wheat flour (10.5–11% protein) with water (50%, flour basis), yeast (1%), and sugar (2%). Pork filling with 30/70 fat-to-lean ratio was hydrated with 20% water. Each assembled bun weighed 60 ± 2 g with a diameter of 6.0 ± 0.2 cm and a height of 4.0 ± 0.1 cm. Proofing was conducted at 35 °C and 75% relative humidity for 30 min until the dough expanded 1.4–1.5 times. Steaming was carried out at 100 ± 1 °C on a rack 4 cm above boiling water. All samples were prepared in a single batch, and physical parameters were strictly controlled, with variation ≤ 3%. Steaming was performed in a closed laboratory steamer preheated to boiling to maintain a saturated steam environment throughout the process.

Baozi was then prepared by filling the dough with the standardized pork filling. The ratio of dough to filling was 3:2. After assembling, the buns were steamed in the steamer for 3, 6, 9, 12, 15, and 18 min, respectively, and then allowed to stand at room temperature for 10 min. Steamed buns were subsequently grouped according to experimental requirements. The maturity state of the buns was assessed by trained professionals in the field.

### 2.3. Specific Volume Measurement

Samples were steamed for durations of 0, 3, 6, 9, 12, 15, and 18 min, and then weighed using a precision balance with an accuracy of 0.01 g. The millet volume displacement method was employed to measure the volume of the stuffed buns, with results accurate to within 1 mL. The formula for a given number of buns is as follows [15]:

SV = V/M(1)

where SV is the specific volume of bun (mL/g); V is the volume of bun, mL; and M is the mass of bun, g.

### 2.4. Texture Properties of Stuffed Bun

On the carrier table, the complete buns were placed with varying steaming periods, and the samples were averaged over three measurements. Measurement parameters (TA-XT Plus, Stable Micro Systems Ltd., Godalming, UK): P/50R probe was utilized with test, pre-test, and post-test rates of 5.0 mm/s, 1.0 mm/s, and 5.0 mm/s, respectively. Also employed were 35% compression, a 5 g trigger force, and a 5 s gap between two compression times [16]. The texture analyzer was calibrated prior to measurement according to the manufacturer’s instructions. Hardness was recorded in grams (g), and mastication was calculated as the product of hardness, cohesiveness, and springiness.

### 2.5. Color of Stuffed Bun

The apparent color of the frozen buns was measured using a colorimeter (CR-400, Konica Minolta, Osaka, Japan) after the buns were steam-cooked for one hour and then allowed to cool to room temperature [17]. The colorimeter was calibrated using a standard white plate before measurement. L* (luminance, 0 white to 100 black), a* (red-green, +direction change indicates an increase in red, −direction change indicates an increase in green), and b* (yellow-blue, +direction change indicates an increase in yellow, − direction change indicates an increase in blue) values are analyzed. The tests are repeated at least 3 times.

### 2.6. Differential Scanning Calorimetry (DSC)

After freeze-drying the stuffed bun dough with different steaming time through a 100 mesh sieve, 15.00 ± 1 mg of the sample was accurately weighed and laid flat in a crucible. Seal the crucible with a crucible lid and press the crucible with a tablet presser to ensure good sealing of the crucible. The crucible was allowed to stand at room temperature for one day and then put into the DSC tester (Q2000, TA Instruments, New Castle, DE, USA) for testing [18]. Temperature and enthalpy calibration were performed using standard reference materials according to the manufacturer’s protocol. The heating rate was 5 °C/min, and the temperature was programmed to increase from 20 °C to 100 °C. The samples were analyzed using the self-contained thermodynamic analysis software to obtain the gelation onset temperature (T_0_), peak temperature (T_P_), termination temperature (T_C_), and enthalpy (ΔH) of the samples.

### 2.7. Gelatinization Degree Determination

The dough of stuffed bun skin samples with different steaming times was freeze-dried and crushed under vacuum. 1.000 g of the sample was taken into two 250 mL conical flasks, labeled B1 and B2, while another conical flask, labeled B0, was used as a blank control without adding the sample. Fifty milliliters of distilled water were added to each flask and mixed thoroughly. The flask B1 was heated on an electric stove until slightly boiling for 20 min, with continuous shaking to prevent drying, and then rapidly cooled to room temperature.

To assess starch gelatinization and structural changes, microbial α-amylase (100 U/mL, Wanbang Chemical Technology Co., Ltd., Zhengzhou, China) was added to flasks B0, B1, and B2 at a final concentration of 1 g/100 mL. The reaction was carried out in an acetate buffer at pH 6.0 and 37 °C for 1 h, with continuous shaking. The reaction was terminated by adding 2 mL of 1 M hydrochloric acid to each flask. The mixtures were then diluted to 100 mL with distilled water and filtered.

Separately, 10 mL of filtrate from each sample was transferred into 250 mL iodine measuring flasks. Immediately, 10 mL of 0.05 mol/L iodine solution and 18 mL of 0.1 mol/L sodium hydroxide solution were added. The flasks were stoppered, kept in the dark for 15 min, and then 2 mL of 10% sulfuric acid solution was added. The remaining iodine was titrated with 0.05 mol/L sodium thiosulfate solution until colorless. The degree of pasting (gelatinization) was calculated as follows:

Degree of pasting = (B_0_ − B_2_)/(B_0_ − B_1_) × 100% (2)

where: B_0_ is the volume of sodium thiosulfate consumed in titrating the blank sample, mL; B_1_ is the volume of sodium thiosulfate consumed in the samples with different steaming times, mL; B_2_ is the volume of sodium thiosulfate consumed in titrating the samples when steaming did not start, mL.

### 2.8. X-Ray Diffraction of Stuffed Bun Skin

The freeze-dried and ground stuffed buns skin sample was placed in the rectangular hole of the aluminum sheet for tablet pressing. The X-ray test conditions are as follows: Scanning speed is 4°/min, scanning area is 5° to 50°, and sampling step width is 0.02°.

### 2.9. Low-Field Nuclear Magnetic Resonance (LF-NMR)

Water mobility and the distribution of steamed buns ware measured by using a NMR Analyzer (MesoMR23-040H-I, Niumag Analytical Instrument Corporation, Suzhou, China). Stuffed buns with different steaming times were taken in the NMR tube and placed in the permanent magnetic field RF center for the Car-Percell Meiboom-Gill (CPMG) pulse series test, and the proton induced decay curves were recorded to determine the relaxation time of the samples, T_2_. The parameters of the measurement samples were set up as follows: waiting time TW = 500 ms, echo time TE = 0.2 ms, cumulative number of times 4, frequency SW = 250 kHz, number of echoes 5000, number of samples 250,022. Using the sirt algorithm in the T2_FitFrm software (embedded system software, Niumag Corporation, Suzhou, China) to process and fit the sampled data from the CPMG series, the T2 value, the spectrogram, and the corresponding amplitude of the proton signal of each sample can be obtained. Each sample was measured in triplicate [19].

### 2.10. Temperature Measurement of Each Layer

The steamed buns were placed in the top steamer once the water reached a rolling boil. A thermocouple was inserted at three positions within each bun to capture the temperature distribution:

Center—at the geometric center of the bun (r = 0 cm, h = 2 cm);

Mid-radius—1.5 cm from the center along the radial direction;

Near bottom—0.7 cm above the bottom surface of the bun.

Temperature readings were recorded every 3 min throughout the steaming process. Each measurement was repeated three times, and the average values were calculated. These positions were selected to represent the temperature gradient from the core to the outer layers, allowing for accurate monitoring of heat transfer and assessment of bun maturity during steaming.

### 2.11. Model Initial Conditions and Boundary Conditions

A two-dimensional axisymmetric heat transfer model was established in COMSOL Multiphysics (v5.6, COMSOL AB, Stockholm, Sweden) to simulate the transient temperature evolution of the stuffed bun during the steaming process. The axisymmetric geometry was adopted because the bun exhibits an approximately rotationally symmetric structure, which allows a significant reduction in computational cost while preserving the essential thermal characteristics of the system.

The simulation focused on transient heat conduction within the bun, and moisture transport, phase change, and structural deformation were not explicitly coupled in the current model. This simplification was adopted because the primary objective of the computational fluid dynamics modeling in this study was to capture the spatiotemporal temperature distribution and heating rate evolution during steaming, which are directly related to starch gelatinization and protein denaturation and thus critical for evaluating the cooking degree of the product. The model therefore serves as a first-order thermal approximation to support experimental observations rather than a fully coupled multi-physics prediction. It should be noted that the present model assumes constant thermophysical properties and does not explicitly consider moisture migration or heat-moisture coupling during steaming.

The initial temperature of the bun was uniformly set to 25 °C, corresponding to room temperature prior to steaming. Saturated steam conditions were assumed during the entire steaming process, and the external surfaces of the bun were subjected to a constant temperature boundary condition of 100 °C, representing the saturated steam environment. Heat transfer within the bun was governed by the transient heat conduction equation:

ρ · Cp · (∂T/∂t) = ∇ · (k∇T)

where ρ is density, Cp is specific heat capacity, k is thermal conductivity, and T is temperature. Thermal properties of the bun were assumed to be homogeneous and constant, and values were adopted from literature reports on wheat-based dough systems.

The bottom surface in contact with the steamer tray was assumed to be thermally insulated, as heat transfer was dominated by steam condensation on the exposed surfaces. Convective heat transfer resistance at the steam-bun interface was neglected under the assumption of saturated steam conditions, where surface temperature rapidly approaches the steam temperature.

A physics-controlled mesh was applied to ensure numerical stability and computational efficiency. Mesh refinement tests were conducted to confirm that further mesh densification resulted in negligible changes in the simulated temperature profiles. A time-dependent solver was used with adaptive time stepping to accurately capture the rapid temperature rise during the early steaming stage.

It should be noted that the current model does not account for moisture migration, pore formation, or volumetric expansion occurring during steaming, which may contribute to discrepancies between simulated and experimental temperatures at later stages. Nevertheless, the model effectively captures the overall heating trends and spatial temperature gradients within the bun, providing valuable insight into the thermal behavior during the steaming process.

### 2.12. Data Analysis

All experiments were conducted in triplicate (*n* = 3), and results are expressed as mean ± standard deviation. Statistical analysis was performed using Origin 2023 and IBM SPSS Statistics 26. One-way analysis of variance (ANOVA) was applied to evaluate the effect of steaming time on measured parameters. When significant differences were detected, mean comparisons were conducted using Tukey’s multiple comparison test. Differences were considered statistically significant at *p* < 0.05. In tables, different superscript letters within the same column indicate significant differences among treatments.

## 3. Results and Discussion

### 3.1. Specific Volume Change at Different Steaming Time

**Figure 1:**
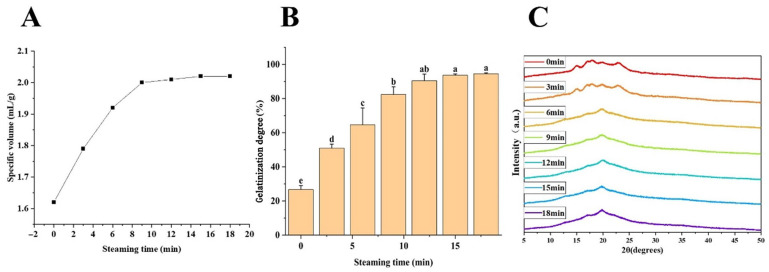
(**A**) The specific volume change of stuffed bun during steaming. (**B**) Gelatinization degree changes with steaming time. (**C**) XRD pattern of stuffed bun with different steaming time. Different lowercase letters (a–e) in panels B indicate significant differences (*p* < 0.05).

Specific volume is one of the most intuitive indicators for evaluating the quality of stuffed buns. Figure 1A illustrates the changes in specific volume during the steaming process. As steaming time increased, the specific volume of the buns showed an overall upward trend. A rapid increase in volume was observed during the first 6 min of steaming, followed by a reduced growth rate between 6 and 9 min, after which the specific volume gradually stabilized. The specific volume reached 2.01 mL/g at 9 min, and further prolongation of steaming time did not result in a significant increase, indicating that the expansion of the bun structure was largely completed by this stage.

### 3.2. Texture Characteristics at Different Steaming Time

Texture reflects the sensory perception arising from the material composition and structural properties of food and plays a critical role in consumer acceptance during mastication and swallowing. Texture profile analysis (TPA) was used to characterize the textural properties of steamed buns, including elasticity, cohesiveness, chewiness, resilience, and hardness. The textural parameters of buns steamed for different durations are summarized in Table 1. As shown in Table 1, the textural properties of unsteamed buns (0 min) were significantly different from those of steamed samples (*p* < 0.05). With increasing steaming time, elasticity, cohesiveness, and resilience increased markedly during the first 0–12 min. This behavior can be attributed to rapid water absorption, starch gelatinization, partial starch hydrolysis, and protein structural rearrangement under high-temperature and high-humidity conditions, leading to the formation of a more uniform and stable network structure. After 12 min of steaming, no significant changes were observed in elasticity, cohesiveness, resilience, or chewiness, suggesting that the internal structure of the buns had reached a relatively stable state.

### 3.3. The Apparent Color of Stuffed Buns at Different Steaming Times

Unlike baked products that typically exhibit a dark brown surface, a bright white appearance is an important quality attribute for steamed buns. As shown in Table 2, unsteamed buns exhibited relatively low brightness (L* = 48.09), with a red-green value (a*) of 1.08 and a yellow-blue value (b*) of 16.36.

With increasing steaming time, the brightness value (L*) increased significantly (*p* < 0.05), while both a* and b* values decreased significantly (*p* < 0.05), indicating that steaming caused the buns to become brighter and shift toward greener and bluer tones. The total color difference (ΔE) followed a trend similar to that of L*. However, although clear color differences were observed between unsteamed and steamed buns, the differences among buns steamed for different durations were relatively small. Therefore, apparent color is not a sensitive indicator for distinguishing maturity levels during the steaming process.

### 3.4. The Thermodynamics of Stuffed Bun Pastry with Different Steaming Time Is Obtained by DSC

The thermal properties of stuffed bun dough with different steaming times were analyzed using differential scanning calorimetry (DSC), and the results are presented in Table 3. As steaming time increased, the onset temperature (T_0_), peak temperature (T_p_), conclusion temperature (T_c_), and gelatinization enthalpy (ΔH) all showed a decreasing trend.

During the initial 0–9 min of steaming, the central temperature of the buns increased rapidly, accompanied by a decrease in the onset and conclusion gelatinization temperatures from 55.7 °C and 62.19 °C to 50.9 °C and 60.62 °C, respectively. No significant changes in gelatinization temperatures were observed between 9 and 18 min. This phenomenon may be attributed to starch hydrolysis and molecular rearrangement under high-temperature and high-humidity conditions, resulting in a more thermally stable starch structure. As the molecular structure becomes more stable, less energy is required for gelatinization, leading to a decrease in ΔH with prolonged steaming time. These results indicate that the major starch gelatinization process was largely completed within the first 9 min of steaming.

### 3.5. The Gelatinization Degree of Stuffed Bun Skin Changes with Different Steaming Time

The changes in the gelatinization degree of stuffed bun skin during steaming are shown in Figure 1B. When the steaming time was 0 min, the gelatinization degree of the dough was relatively low (26.73%). With increasing steaming time, the gelatinization degree increased rapidly and reached 82.43% at 9 min. From 9 to 12 min, the increase in gelatinization degree slowed, and only a minor increase (approximately 3.2%) was observed between 12 and 15 min. When the steaming time was extended to 18 min, the gelatinization degree approached a plateau at approximately 95% and remained nearly unchanged thereafter.

During the steaming process, the continuous increase in temperature and moisture availability enhanced molecular vibration within starch granules, leading to the disruption of hydrogen bonds and increased interaction between starch molecules and water. As a result, the crystalline regions were gradually reduced, promoting starch gelatinization and paste formation, consistent with previous reports [20].

### 3.6. X-Ray Diffraction Analysis on the Crystallization of Stuffed Bun Skin

Figure 1C presents the X-ray diffraction (XRD) patterns of stuffed bun skin at different steaming times. The diffraction patterns of samples steamed for 0 and 3 min exhibited typical A-type starch characteristics, with prominent peaks at 15°, 17°, 18°, and 23° (2θ). As steaming proceeded, starch gelatinization intensified, and these characteristic diffraction peaks completely disappeared after 6 min of steaming.

At 6 min, the gelatinization degree of the bun skin reached approximately 65%, which is consistent with previously reported XRD results for wheat starch at similar gelatinization levels [21]. Overall, the crystallinity of the stuffed bun skin decreased progressively with increasing steaming time and temperature, indicating a gradual transition from an ordered crystalline structure to a more amorphous state.

### 3.7. Moisture Migration of Stuffed Buns at Different Steaming Times

The distribution and mobility of moisture within stuffed buns play a critical role in determining their final quality. Low-field nuclear magnetic resonance (LF-NMR) was used to analyze moisture migration during steaming, with the transverse relaxation time (T_2_) reflecting the mobility of different water populations. Smaller T_2_ values correspond to more tightly bound water and lower mobility, whereas larger T_2_ values indicate increased water mobility [22].

**Figure 2:**
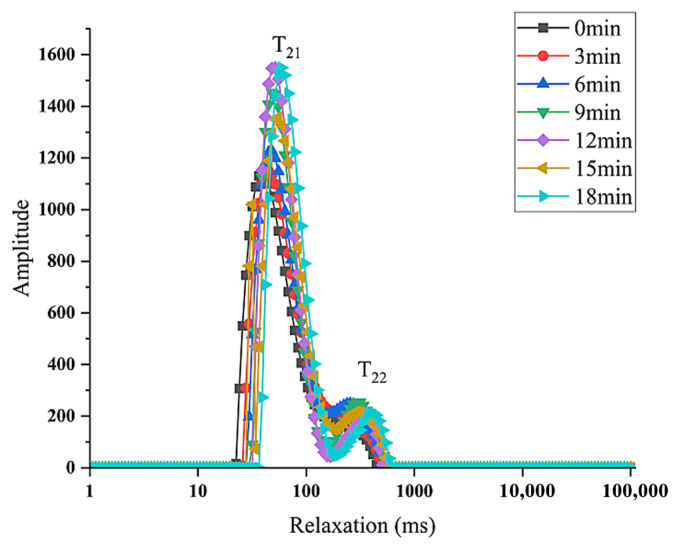
The change of T2 relaxation time of the whole stuffed bun with different steaming times.

As shown in Figure 2, two major relaxation peaks, T_21_ and T_22_, were observed. T_21_ represents bound water tightly associated with starch and gluten proteins, characterized by low mobility, while T_22_ corresponds to weakly bound water with relatively higher mobility [23]. Bound water constituted the predominant water fraction in all samples. The proportion of bound water increased steadily from 0 to 12 min of steaming, decreased between 12 and 15 min, and then increased again from 15 to 18 min. This behavior may be attributed to protein denaturation and structural contraction during heating, which temporarily expelled bound water and converted it into weakly bound water. No obvious macroscopic water migration from the filling to the dough, or vice versa, was observed during steaming. The observed changes in water distribution were mainly associated with starch gelatinization and protein hydration within the dough matrix.

In addition, a noticeable rightward shift in the T_2_ relaxation peaks was observed with increasing steaming time, indicating enhanced water mobility within the buns as the internal structure became progressively loosened during heating.

### 3.8. Model Verification

**Figure 3:**
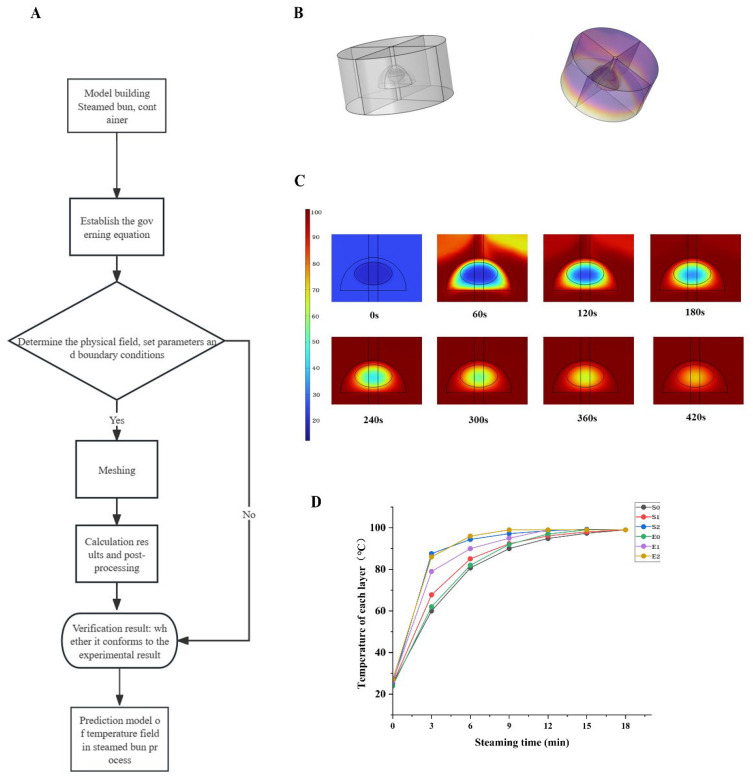
(**A**) Model design route. (**B**) Model 3D image. (**C**) In the process of computer simulation, the temperature field of the stuffed bun in each time period changes. (**D**) For the temperature changes of each layer of the stuffed bun at different steaming times, S_0_, S_1_, and S_2_ are the temperature at the filling distance of 1 cm and 2 cm respectively from the filling distance of the stuffed bun. E_0_, E_1_, and E_2_ are the filling temperature of the bun and the temperature change at the distance of 1 cm and 2 cm from the filling center during the experiment.

The schematic design of the temperature measurement and modeling approach is shown in Figure 3A. Thermocouples were used to monitor temperature changes at the center of the bun and at distances of 1 cm and 2 cm from the center during steaming. As shown in Figure 3D, the initial temperature at the center of the bun was the lowest, while the temperature at 2 cm from the center was the highest, likely due to closer proximity to the steam environment.

Heat transfer within the stuffed bun during steaming was dominated by heat conduction. As steaming time increased, temperatures at all monitored positions exhibited an overall upward trend. A rapid temperature increase was observed during the first 0–3 min, followed by a slower increase from 3 to 12 min. By 15 min of steaming, the temperatures of all layers approached approximately 99 °C. Among the monitored positions, the temperature at 2 cm from the center increased most rapidly, followed by that at 1 cm, while the center exhibited the slowest heating rate.

The temperature field within the bun was further simulated using numerical modeling, as illustrated in Figure 3C, with a three-dimensional representation shown in Figure 3B. As demonstrated in Figure 3D, the simulated temperature trends were in good agreement with experimental measurements. The experimentally measured temperatures were slightly higher than the simulated values, which may be attributed to the formation of pores within the bun during steaming. Pore formation can enhance heat transfer efficiency, an effect not explicitly considered in the numerical model. The omission of moisture–heat coupling may partially contribute to the slight deviation between simulated and experimental temperatures at later steaming stages.

At a steaming time of 9 min, the simulated temperatures at the center (S_0_), 1 cm (S_1_), and 2 cm (S_2_) from the center were 90.23 °C, 92.35 °C, and 97.17 °C, respectively, while the corresponding experimental values (E_0_, E_1_, E_2_) were 92 °C, 95 °C, and 99 °C. The close agreement between simulated and experimental results confirms the feasibility and reliability of the proposed temperature model. Based on the combined results of starch gelatinization, structural stabilization, and temperature evolution, major quality transformation was completed when the core temperature reached approximately 90–93 °C. This temperature range is sufficient to ensure both microbial safety and adequate gelatinization under the present conditions.

## 4. Conclusions

The dynamic changes of multiple quality indicators during the steaming process of stuffed buns were systematically investigated, and suitable characteristic parameters were evaluated for monitoring bun ripening under controlled steaming conditions. Three main findings were obtained.

First, color parameters showed limited sensitivity to steaming duration. Although chroma values clearly distinguished unsteamed buns from steamed samples, no significant differences were observed among buns steamed for different durations (*p* > 0.05). Therefore, instrumental color parameters were not considered suitable indicators for monitoring steaming maturity in this study.

Second, moisture-related characteristics exhibited pronounced dynamic changes during steaming. With increasing steaming time, the relaxation times gradually shifted to the right, indicating enhanced water mobility within the bun matrix. A transient decrease in moisture content was observed at 12–15 min, followed by a subsequent increase after prolonged steaming. Combined with the stabilization of internal central temperature (approximately 93 °C at 9–12 min), threshold ranges of T_21_ and T_22_ were empirically identified as potential indicators for moderate steaming under the experimental conditions applied. Accordingly, a core temperature of approximately 90–93 °C is recommended as a practical target for maturity monitoring. In parallel, gelatinization degree, specific volume, and XRD patterns exhibited substantial changes during the initial 0–9 min steaming stage, followed by a plateau thereafter, suggesting that major structural transformations were largely completed within this period.

Third, a numerical temperature simulation model was established to describe heat transfer during the steaming process and to assist in maturity prediction. Based on the combined evolution of temperature and quality attributes, the steaming process could be operationally divided into three stages: an early stage (0–6 min), suitable for partially cooked or quick-frozen buns; an intermediate stage (9–12 min), corresponding to proper steaming; and a prolonged stage (≥15 min), associated with over-steaming. It should be noted that this stage classification represents an empirical framework derived from the present experimental conditions rather than a universal standard.

This study demonstrates that the combination of internal temperature and steaming time can effectively distinguish different steaming states of stuffed buns, providing a practical basis for process monitoring and control. However, the applicability of the proposed thresholds and classifications may be influenced by bun formulation, size, filling composition, and steaming system. Future studies should focus on validating the proposed indicators across different formulations and industrial-scale steaming conditions, as well as incorporating moisture transport and structural evolution into coupled multi-physics models to further improve prediction accuracy.

## Figures and Tables

**Table 1:** The change of texture characteristics of stuffed bun in the process of steaming.

Steaming Time	Elasticity (-)	Cohesiveness (-)	Mastication (g)	Resilience (-)
0 min	0.32 ± 0.22 ^c^	0.26 ± 0.06 ^c^	134.28 ± 4.03 ^d^	0.06 ± 0.04 ^d^
3 min	0.62 ± 0.04 ^b^	0.58 ± 0.03 ^b^	1334.41 ± 52.31 ^c^	0.19 ± 0.08 ^c^
6 min	0.69 ± 0.07 ^b^	0.59 ± 0.09 ^b^	2429.96 ± 72.90 ^b^	0.21 ± 0.11 ^bc^
9 min	0.71 ± 0.04 ^b^	0.62 ± 0.03 ^b^	2733.53 ± 81.71 ^ab^	0.24 ± 0.05 ^b^
12 min	0.84 ± 0.11 ^a^	0.71 ± 0.01 ^a^	2836.81 ± 84.55 ^ab^	0.32 ± 0.00 ^a^
15 min	0.87 ± 0.04 ^a^	0.68 ± 0.02 ^a^	3249.48 ± 97.48 ^ab^	0.30 ± 0.03 ^a^
18 min	0.85 ± 0.06 ^a^	0.68 ± 0.05 ^a^	3480.88 ± 101.23 ^a^	0.29 ± 0.09 ^a^

Note: All data were reported as mean and standard deviations (*n* = 3). Values with different superscripts in the same column are significantly different (*p* < 0.05).

**Table 2:** Changes in the apparent color of stuffed buns during steaming.

Steaming Time	L*	a*	b*	ΔE
0 min	48.09 ± 0.22 ^e^	1.08 ± 0.01 ^a^	16.36 ± 0.29 ^a^	46.8 ± 0.20 ^a^
3 min	86.77 ± 0.22 ^ab^	−1.16 ± 0.02 ^b^	14.35 ± 0.30 ^c^	12.47 ± 0.01 ^c^
6 min	85.85 ± 0.70 ^cd^	−1.22 ± 0.02 ^b^	13.44 ± 0.08 ^d^	11.73 ± 0.21 ^d^
9 min	86.20 ± 0.10 ^bc^	−1.33 ± 0.05 ^c^	13.48 ± 0.06 ^d^	11.61 ± 0.16 ^d^
12 min	85.44 ± 0.12 ^d^	−1.92 ± 0.06 ^d^	14.35 ± 0.12 ^c^	13.19 ± 0.16 ^b^
15 min	85.3 ± 0.16 ^d^	−2.19 ± 0.04 ^e^	14.76 ± 0.08 ^b^	13.19 ± 0.12 ^b^
18 min	86.95 ± 0.22 ^a^	−2.23 ± 0.1 ^e^	14.31 ± 0.17 ^c^	12.34 ± 0.30 ^c^

Note: All data were reported as mean and standard deviations (*n* = 3). Values with different superscripts in the same column are significantly different (*p* < 0.05). ΔE values represent color differences relative to the unsteamed bun (0 min), which was defined as the reference sample.

**Table 3:** Thermodynamic parameters of stuffed bun crust with different steaming time.

Steaming Time	T_0_ (°C)	T_P_ (°C)	T_C_ (°C)	ΔH
0 min	55.7 ± 0.45 ^a^	57.6 ± 0.61 ^a^	62.19 ± 0.07 ^a^	0.56 ± 0.01 ^a^
3 min	52.5 ± 0.73 ^b^	54.8 ± 0.71 ^b^	62.03 ± 0.08 ^a^	0.49 ± 0.01 ^b^
6 min	51.2 ± 0.41 ^c^	53.2 ± 0.37 ^c^	61.12 ± 0.08 ^b^	0.39 ± 0.06 ^c^
9 min	50.9 ± 0.06 ^cd^	52.9 ± 0.17 ^c^	60.62 ± 0.16 ^c^	0.34 ± 0.02 ^d^
12 min	50.6 ± 0.09 ^cd^	52.4 ± 0.24 ^c^	60.49 ± 0.19 ^cd^	0.29 ± 0.00 ^de^
15 min	50.4 ± 0.16 ^d^	52.6 ± 0.15 ^c^	60.32 ± 0.11 ^d^	0.27 ± 0.01 ^e^
18 min	50.2 ± 0.06 ^d^	52.4 ± 0.36 ^c^	60.30 ± 0.23 ^d^	0.24 ± 0.01 ^e^

Note: All data were reported as mean and standard deviations (*n* = 3). Values with different superscripts in the same column are significantly different (*p* < 0.05). T_0_, T_P_ and T_C_ represent the gelation starting temperature, peak temperature, and end temperature respectively, and ΔH represents the enthalpy change.

## Data Availability

The data presented in this study are available in the article. Further inquiries can be directed to the corresponding author.
